# A new mouse SNP genotyping assay for speed congenics: combining flexibility, affordability, and power

**DOI:** 10.1186/s12864-021-07698-9

**Published:** 2021-05-24

**Authors:** Kimberly R. Andrews, Samuel S. Hunter, Brandi K. Torrevillas, Nora Céspedes, Sarah M. Garrison, Jessica Strickland, Delaney Wagers, Gretchen Hansten, Daniel D. New, Matthew W. Fagnan, Shirley Luckhart

**Affiliations:** 1 Institute for Bioinformatics and Evolutionary Studies (IBEST), University of Idaho, Moscow, ID 83844 USA; 2 Department of Entomology, Plant Pathology and Nematology, University of Idaho, Moscow, ID 83844 USA; 3 Department of Biological Sciences, University of Idaho, Moscow, ID 83844 USA

**Keywords:** Speed congenics, Illumina, Next generation sequencing, Allegro targeted genotyping, Single primer enrichment technology, Bioinformatic pipeline

## Abstract

**Background:**

Speed congenics is an important tool for creating congenic mice to investigate gene functions, but current SNP genotyping methods for speed congenics are expensive. These methods usually rely on chip or array technologies, and a different assay must be developed for each backcross strain combination. “Next generation” high throughput DNA sequencing technologies have the potential to decrease cost and increase flexibility and power of speed congenics, but thus far have not been utilized for this purpose.

**Results:**

We took advantage of the power of high throughput sequencing technologies to develop a cost-effective, high-density SNP genotyping assay that can be used across many combinations of backcross strains. The assay surveys 1640 genome-wide SNPs known to be polymorphic across > 100 mouse strains, with an expected average of 549 ± 136 SD diagnostic SNPs between each pair of strains. We demonstrated that the assay has a high density of diagnostic SNPs for backcrossing the BALB/c strain into the C57BL/6J strain (807–819 SNPs), and a sufficient density of diagnostic SNPs for backcrossing the closely related substrains C57BL/6N and C57BL/6J (123–139 SNPs). Furthermore, the assay can easily be modified to include additional diagnostic SNPs for backcrossing other closely related substrains. We also developed a bioinformatic pipeline for SNP genotyping and calculating the percentage of alleles that match the backcross recipient strain for each sample; this information can be used to guide the selection of individuals for the next backcross, and to assess whether individuals have become congenic. We demonstrated the effectiveness of the assay and bioinformatic pipeline with a backcross experiment of BALB/c-IL4/IL13 into C57BL/6J; after six generations of backcrosses, offspring were up to 99.8% congenic.

**Conclusions:**

The SNP genotyping assay and bioinformatic pipeline developed here present a valuable tool for increasing the power and decreasing the cost of many studies that depend on speed congenics. The assay is highly flexible and can be used for combinations of strains that are commonly used for speed congenics. The assay could also be used for other techniques including QTL mapping, standard F2 crosses, ancestry analysis, and forensics.

**Supplementary Information:**

The online version contains supplementary material available at 10.1186/s12864-021-07698-9.

## Background

The development of methods to create “congenic” mice has led to substantial advances in our understanding of the functions of genes and mutations (e.g. [1, 2],). These methods involve transferring the gene or mutation of interest to a standard genetic background to eliminate the impact of confounding genetic interactions that could influence the phenotype. Traditionally, the development of a congenic background has been accomplished by backcrossing a mutant line with a standard inbred laboratory strain of the preferred genetic background. Although popularity has grown for new genome editing techniques that transfer genetic content to a new background without the need for backcrossing, such as the Cas9 based strategies, these techniques have disadvantages compared to traditional approaches, including off-target effects and limitations in the length of the mutation that can be transferred [3–5]. A major disadvantage of the traditional congenic approach, however, is the length of time required for backcrossing; this approach required ten backcross generations, which can take up to 3 years. The development of “speed congenics” substantially sped up the traditional congenics approach by cutting in half the number of required backcross generations [6, 7]. Speed congenics uses genetic markers to identify backcross offspring with the highest levels of ancestry for the desired genetic background. By preferentially selecting these individuals for the next backcross step, the number of generations required to develop congenic mice can be reduced from ten to five.

Speed congenics has been used for more than two decades, and rapid advances in genetic analysis technologies have led to steady improvements in the power, efficiency, and cost-effectiveness of this approach. These advances have led to the discovery of large numbers of genetic markers that can differentiate commonly used backcross strains, thus improving the power and efficiency of speed congenics by increasing the density of informative genetic markers across the genome. In addition, technological advances have led to improvements in the efficiency and cost of methods for generating genetic data for these markers. Initially, speed congenics relied on microsatellite markers (also known as simple sequence length polymorphisms, or SSLPs), but most approaches now rely on single nucleotide polymorphism markers (SNPs) due to the increased efficiency of genotyping techniques designed around these markers [8, 9]. Most SNP-based assays for speed congenics employ chip or array technologies, typically using around 150 genome-wide diagnostic SNPs that distinguish the two backcross strains. These assays require a separate set of diagnostic SNPs for each unique combination of backcross strains. Other SNP arrays have been developed to survey genetic variation across multiple strains and substrains using many thousands of SNPs (e.g., the Mouse Diversity Array [10] and the Mouse Universal Genotyping Arrays or MUGAs [9, 11]). However, these arrays are expensive and provide data from many more sites than is typically required for speed congenics experiments. Furthermore, these chip and array techniques rely on specialized equipment found in relatively few research labs, thereby leading most researchers to outsource SNP genotyping for speed congenics.

Thus far, speed congenics genotyping approaches have not taken full advantage of “next generation” high throughput DNA sequencing technologies, which have the potential to increase the flexibility, affordability, and power of genotyping. Although these technologies have been used to characterize the ancestry of backcross offspring by sequencing whole genomes and whole exomes, those approaches are cost-prohibitive and require complex data analysis with extensive computational resources [12]. Rather than sequencing whole genomes or whole exomes, high throughput sequencing can be harnessed to generate sequence data for hundreds of targeted SNPs that are informative for speed congenics; this approach can be fast and inexpensive, with much lower demands for computational resources and much less complexity in data analysis.

Here we developed a SNP genotyping assay for speed congenics that takes advantage of high throughput sequencing technology and utilizes 1640 SNPs that are diagnostic across a wide variety of commonly used laboratory mouse strains and substrains. The assay uses the Allegro Targeted Genotyping method developed by Tecan (Mannedorf, Switzerland) and relies on Illumina sequencing platforms (Illumina, Inc., San Diego, USA) that are commonly available in core labs. The assay is designed so that most strain combinations should have at least 300 diagnostic SNPs, with an average of 549 diagnostic SNPs across strain pairs, providing a high level of flexibility for use across many strain combinations. The assay can also be easily modified to incorporate additional informative SNPs for custom experiments, such as for backcrosses using closely related substrains. We also developed a bioinformatic pipeline to analyze the sequence data, including SNP genotyping and calculation of the percentage of alleles that match the backcross recipient strain for each sample. We tested the performance of the assay on three commonly used backcross strains or substrains from multiple sources, and found the assay to have a high density of genome-wide SNPs for distinguishing strains BALB/c and C57BL/6J (807–819 SNPs) and a sufficient density of SNPs for distinguishing the closely related substrains C57BL/6J and C57BL/6N (123–139 SNPs). We expect the flexibility and affordability of this SNP genotyping assay to make it a powerful and practical tool for many projects that depend on speed congenics.

## Methods

### Assay design

We used prior published studies to identify SNPs for our genotyping assay that would be informative for speed congenics across a wide range of mouse strain combinations. We chose SNPs from a study that used public databases to identify 1638 SNPs that were evenly distributed across the mouse genome (approximately 1.5 Mb between SNPs) and were polymorphic across 102 inbred and wild-derived inbred mouse strains, with an average of 600 SNPs being diagnostic between each pair of strains, and 97% of pairs having at least 300 diagnostic SNPs [13]. We also selected 141 SNPs known to distinguish the substrains C57BL/6J and C57BL/6NJ from the GigaMUGA, which is a 143,259-probe Illumina Infinium II array designed for distinguishing multiple mouse strains and substrains [9]. The set of SNPs was chosen to strike a balance between a sufficient number of markers to achieve high power and flexibility to distinguish multiple strain combinations, while minimizing the total number of markers to reduce sequencing costs and computational requirements for bioinformatic analysis.

The Allegro Targeted Genotyping method used in our assay implements Single Primer Enrichment Technology, which involves hybridization of custom-designed probes near target SNPs, followed by probe extension, addition of sequencing adapters, and high throughput Illumina sequencing. Probes for the target SNPs were 40 bp long and were custom-designed by Tecan using the UCSC mm10 genome assembly of the C57BL/6J strain (Accession ID GCA_000001305.2) as a reference. Two probes were designed per target SNP, with one probe hybridizing to the plus strand and the other to the minus strand, and each probe hybridizing within 100 bp of the target SNP. For a small number of our target SNPs, probes could not be designed based on the criteria required by Tecan, or initial runs of the genotyping assay resulted in low numbers of sequence reads across samples. For these SNPs, probes were re-designed by extending the design window by 60 bp on each side of the target SNP, and these new probes were added into the panel. The final probe set targeted a total of 1640 SNPs informative for speed congenics, including 1591 on the autosomes and 49 on the X chromosome (Tables S1, S2). The probe set also targeted 29 SNPs on the Y chromosome (Tables S1, S2). Y chromosome SNPs are not typically used for guiding speed congenics experiments, since the majority of the Y chromosome does not recombine and, therefore, ancestry will be known based on the breeding strategy. Y chromosome SNPs, however, could be used for other applications as noted below.

### Laboratory work

Genomic DNA samples were prepared from <5mm^2^ tail biopsies collected from mice that were 10–15 days old (total *n* = 174 mice, with only one biopsy collected per mouse). After biopsy collection, mice were not euthanized and were returned to their cages. Genomic DNA was extracted from biopsies using Qiagen DNeasy Blood and Tissue kits, following the quick step protocol. All procedures were approved by the Institutional Animal Care and Use Committee of the University of Idaho (protocol #IACUC-2020-10). Genomic DNA was quantified using the Quant-iT Picogreen dsDNA assay kit on Molecular Devices SpectraMax Paradigm Multi-Mode Microplate Detection Platform, and this information was used to normalize sample concentrations. Genomic DNA integrity was assessed using agarose gel electrophoresis or the Advanced Analytical Fragment Analyzer (Agilent, Santa Clara California).

We followed the standard manufacturer guidelines for the Allegro Targeted Genotyping library prep, with some modifications to decrease cost. Standard library prep involves enzymatic fragmentation of high molecular weight genomic DNA, followed by ligation of adapters containing a unique barcode (also called an index) for each sample, pooling of samples, probe hybridization and extension, and library amplification. To reduce the cost of library prep and sequencing, we used MagBio HighPrep PCR Clean-up System beads (MagBio Genomics Inc., Maryland, USA) instead of Agencourt AMPure XP Beads (Beckman Coulter, Indiana, USA) for all bead purification steps. In addition, we used custom bead cleaning ratios to generate libraries with longer fragment lengths than the standard protocol (aiming for 400-1000 bp range, peak at 600 bp). Longer fragments allowed sequencing on Illumina MiSeq 2 × 300 sequencing runs, whereas the standard protocol aims to generate libraries with shorter fragments for 2 × 150 runs, usually performed on the Illumina HiSeq or NextSeq. The use of MiSeq reduced the cost of our assay because our application did not require as many reads as would be produced by the more expensive HiSeq or NextSeq runs. However, the libraries from our genotyping assay could have alternatively been sequenced using 2 × 100 or 2 × 150 runs on any Illumina sequencing platform (e.g., MiSeq, HiSeq, NextSeq). A small number of target SNPs would have reduced coverage with these run types because some SNPs are > 100 bp from the beginning of one or both probes; this reduced coverage is expected for 26 SNPs for 2 × 100 runs, and four SNPs for 2x150bp runs. We further reduced cost by sequencing on a partial MiSeq lane (one-quarter lane), allowing cost-sharing of full runs across researchers. Lane-sharing could be implemented on other Illumina sequencing platforms as well, although not all sequencing facilities provide lane-sharing as a service option. We prepared libraries in batches of 48 samples and sequenced each batch on one-quarter of an Illumina MiSeq V3 2 × 300 sequencing run at the Genomics Resources Core at the University of Idaho.

### Bioinformatic analysis: genotyping

**Fig. 1:**
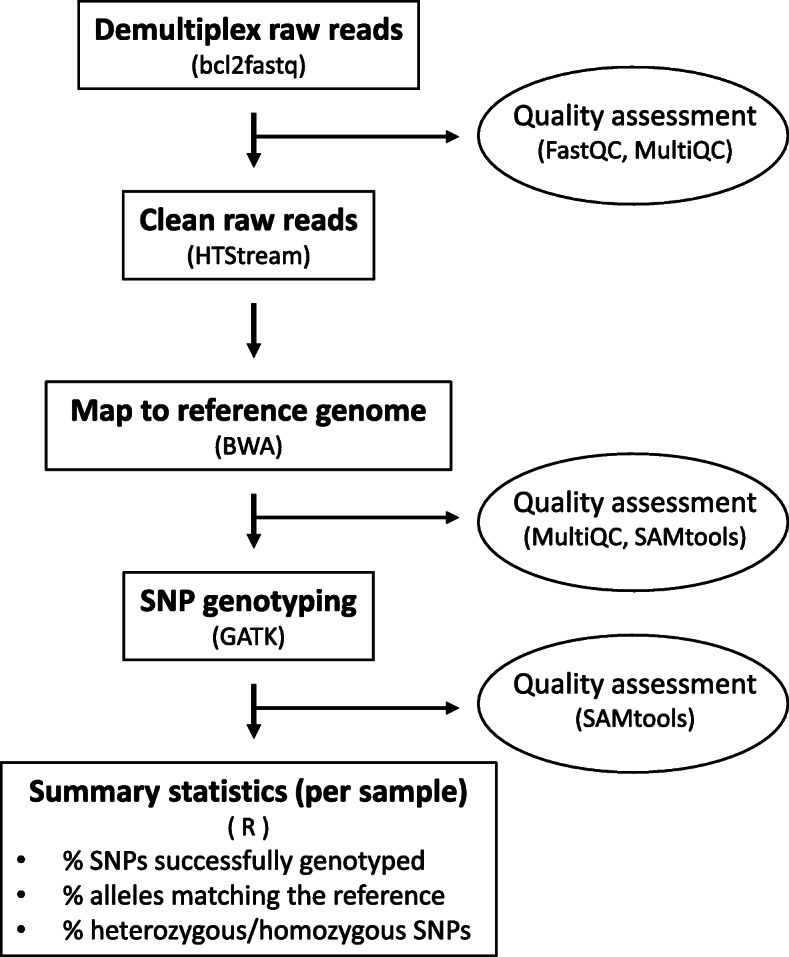
Bioinformatic pipeline for SNP genotyping and generating summary statistics to inform speed congenics experiments. More details on the pipeline can be found at https://github.com/kimandrews/CongenicMouseGenotyping

We developed a bioinformatic pipeline that analyzes the sequence data generated by our assay, producing output that can be easily interpreted to aid in practical decision-making for speed congenics experiments (Fig. 1). The pipeline first demultiplexes sequence reads (separates reads by sample based on unique barcodes) using bcl2fastq v2.20.0.422 (Illumina, Inc), and provides an assessment of sequence quality across samples using FastQC [14] and MultiQC [15]. Reads are then cleaned using HTStream v1.1.0 (https://github.com/s4hts/HTStream/releases/tag/v1.1.0-release) to remove PCR duplicates and adapter sequence, trim probe sequence (i.e., the first 40 bp of each forward read), and remove reads shorter than 90 bp. Cleaned sequence reads are mapped to the reference genome of the backcross recipient strain using BWA v0.7.17 [16], and mapping rates across samples are evaluated using MultiQC. SNP genotyping is conducted using GATK v4.1.3.0 [17] by generating intermediate GVCF files for each sample using HaplotypeCaller, followed by merging of all GVCFs using GenomicsDBImport, and joint genotyping with GenotypeGVCFs. To assess sequencing performance across SNPs for each sample, the number of mapped sequencing reads per sample and SNP are calculated using SAMtools v1.5 [18] with a bed file containing the reference genome locations of the target SNPs, and boxplots are created showing the distribution of the number of mapped sequence reads per SNP for each sample using R v3.6.0 [19].

The pipeline outputs the SNP genotype calls for each sample, as well as a summary of the total percentage of alleles that match the reference allele for the 1640 autosomal and X chromosome SNPs for each sample, and the number and percentage of SNPs with each possible genotype (homozygous for the reference allele, homozygous for the alternate allele, or heterozygous) for each sample. The pipeline also outputs the percentage of SNPs that were successfully genotyped for each sample, to allow easy identification of samples that performed poorly.

### Testing assay performance: genotyping success

To evaluate the quality and consistency of genotyping across samples and SNPs for our custom-designed probe panel, we prepared and sequenced libraries for three batches of 48 samples (total *n* = 144; Table S3), including samples from three mouse strains or substrains that are commonly used in backcross experiments (BALB/c, *n* = 9 libraries from 9 mice); C57BL/6N, *n* = 9 libraries from 6 mice, including one technical replicate for each of three mice; and C57BL/6J, *n* = 12 libraries from 9 mice, including one technical replicate for each of three mice), and samples from multiple generations of backcrosses between these strains (*n* = 114). Library prep, sequencing, and bioinformatic analyses were performed with samples in a blinded format. We evaluated the consistency of sequencing performance across samples by comparing the number of demultiplexed sequence reads for each sample, as well as the number of mapped sequence reads per SNP per sample.

### Testing assay performance: utility for speed congenics

The effectiveness of genotype data for informing backcross experiments lies in the number of diagnostic SNPs, i.e. autosomal and X chromosome SNPs that are homozygous for different alleles between the two strains, and the evenness of the spacing of those SNPs across the genome. To evaluate the effectiveness of our SNP panel for speed congenics for different combinations of strains and substrains, we determined the number and genomic distribution of diagnostic SNPs for backcrosses between two genetically divergent strains (donor BALB/c into recipient C57BL/6J) and two genetically similar substrains (donor C57BL/6N into recipient C57BL/6J) that are commonly used in backcross experiments. To accomplish this, we conducted our genotyping assay for representative mice from BALB/c strains from two sources (BALB/c-AnNHsd from Envigo and BALB/c-IL4/IL13 from The Jackson Laboratory), C57BL/6N strains from two sources (C57BL/6N-Crl from Charles River and C57BL/6N-Hsd from Envigo), and C57BL/6J from one source (The Jackson Laboratory). For each of these strains and sources, we used the results of our genotyping assay for three individual mice with high genotyping success rates (97.1–98.5% of SNPs successfully genotyped) to identify diagnostic SNPs, with the exception of BALB/c-IL4/IL13, for which only two individual mice were available (96.7–97.4% of SNPs successfully genotyped) (Tables 1, S3). For the bioinformatic pipeline, we used the UCSC mm10 C57BL/6J genome assembly as a reference. To identify diagnostic SNPs for each donor strain (assuming the recipient strain is always C57BL/6J), we conducted filtering steps to retain SNPs that consistently genotyped for the donor strain and were homozygous for a different allele than C57BL/6J. We first filtered the SNP panel to remove SNPs that failed to genotype in more than one individual from the donor strain, and then removed SNPs for which any individual from the donor strain was heterozygous or homozygous for the C57BL/6J allele. We conducted this filtering separately for each source of donor strains, since the same strain from different sources can have genetic differences. To examine the spacing across the genome of the diagnostic SNPs for each donor strain, we calculated the number of SNPs per chromosome and the distance between adjacent SNPs on each chromosome for each set of diagnostic SNPs. We also plotted the position of each SNP along each chromosome using the R package chromoMap v0.2 [20].

**Table 1:** Sample sizes and summary statistics comparing strain genotypes against the C57BL/6J reference genome, including the mean, minimum, and maximum number of SNPs that were homozygous for the alternate allele (i.e., not the C57BL/6J allele) as well as mean, minimum, and maximum percentage of C57BL/6J alleles

Strain	Source	Number of homozygous alternate SNPs			% C57BL/6J alleles		
		Mean	Min	Max	Mean	Min	Max
C57BL/6J	Jackson Laboratory (Cat# 000664)	1	1	1	99.9	99.9	99.9
BALB/c-AnNHsd	Envigo	845	840	851	46.8	46.7	46.8
BALB/c-IL4/IL13	Jackson Laboratory (Cat# 015859)	836	830	842	46.9	46.7	47.1
C57BL/6N-Crl	Charles River	142	141	142	91.1	91.1	91.2
C57BL/6N-Hsd	Envigo	126	125	127	92.1	92.0	92.1

We further evaluated the effectiveness of the genotyping assay for speed congenics by using the assay to inform a backcross experiment with one of the donor strains (BALB/c-IL4/IL13, The Jackson Laboratory; Table 1) into C57BL/6J. We initially bred one male of the donor strain with two females of the recipient strain, and three male offspring from this cross were each bred with two females from the recipient strain. We then conducted the genotyping assay for all offspring of both sexes that had the gene of interest, using the bioinformatic pipeline to calculate the percentage congenic alleles across the diagnostic SNPs for each individual. We chose individuals for the next backcross based on which samples had the highest percentage of congenic alleles. For each subsequent backcross, we ran the genotyping assay for all offspring with the gene of interest, choosing the individuals for the next backcross based on the samples with the highest percentage of congenic alleles. We used two to three breeders per generation and performed backcrosses until 99.8% of the congenic strain was achieved in the offspring, following standard congenics practices (e.g. [6, 21, 22],). We chose to genotype all offspring containing the gene of interest at each generation to maximize the effectiveness of the speed congenics approach and thereby minimize the total number of generations required (Table S3) [23].

We also performed bioinformatic analyses to predict the number of diagnostic SNPs for crosses of additional laboratory mouse strains. To accomplish this, we used the genotypes reported in [13] for 102 mouse strains for all SNPs that were shared between that study and our assay (i.e., a total of 1499 SNPs). We calculated the number of predicted diagnostic SNPs for each cross as the number of SNPs with different genotypes between each pair of strains using R v3.6.0.

## Results

### Genotyping performance

**Fig. 2:**
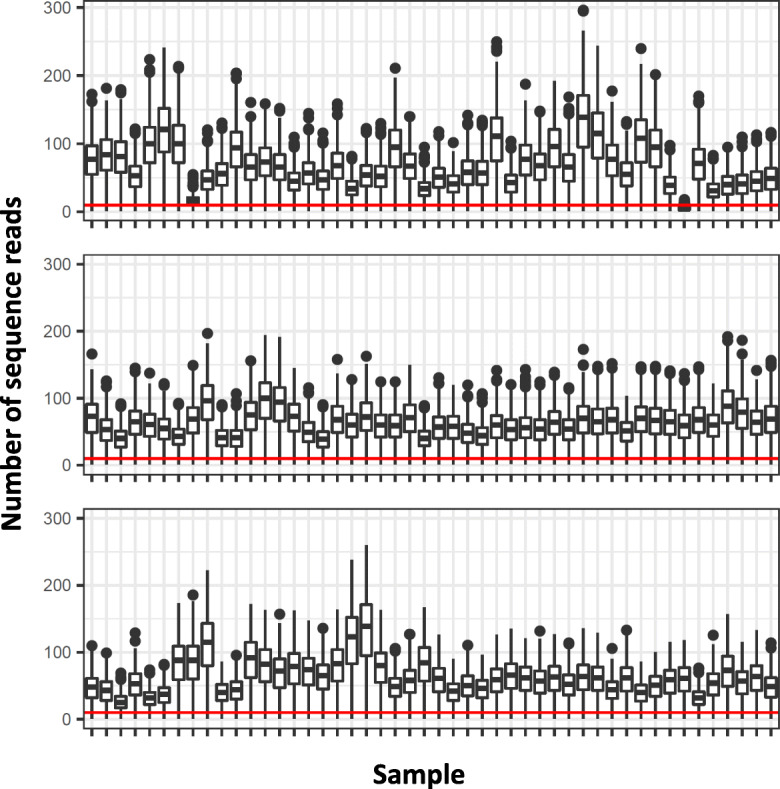
Distributions of the numbers of sequence reads per SNP per sample for each of three batches of 48 samples. The red line occurs at *y* = 10 sequence reads; samples with median values above this line typically have high genotyping success rates

For the three batches of 48 samples that were used to test the genotyping performance of our SNP assay, the total number of demultiplexed sequence reads ranged from 5,290,919 to 7,050,716, and reads were fairly evenly distributed across samples within batches, with mean reads per sample ranging from 110,227 to 146,890 across batches (Table 2). Mapping rates were consistently high across samples and batches, with > 99.5% of reads mapping to the reference genome for each sample. The majority of SNPs had more than ten mapped sequence reads for all samples, except one poor-performing sample in the first batch for which most SNPs had fewer than ten reads (Fig. 2). The number of autosomal SNPs successfully genotyped ranged from 1504 to 1565 across samples, corresponding to 94.5–98.4% of all autosomal SNPs in the panel. The number of X chromosome SNPs successfully genotyped ranged from 46 to 49, corresponding to 93.9–100% of all X chromosome SNPs in the panel. The number of Y chromosome SNPs genotyped for males ranged from 25 to 29, corresponding to 86.2–100% of all Y chromosome SNPs, except for one male sample for which only ten Y chromosome SNPs were genotyped.

**Table 2:** Total number of demultiplexed sequence reads across three batches of 48 samples, and the mean and standard deviation of the number of sequence reads across samples within each batch. St. dev. = standard deviation

Batch	Total	Per sample	
		Mean	St. dev.
1	7,050,716	146,890	64,945
2	5,290,919	110,227	25,151
3	5,412,022	112,750	41,635

### Assay performance for speed congenics

As expected, the majority of SNPs in our C57BL/6J samples were homozygous for C57BL/6J reference alleles, with 99.9% of alleles matching the reference for all samples and replicates (Table 1). For our BALB/c samples, 46.7–47.1% of alleles matched the C57BL/6J reference alleles, and for our C57BL/6N samples, 91.1–92.1% of alleles matched the C57BL/6J reference alleles. Few SNPs were heterozygous for BALB/c or C57BL/6N samples (< 1.4% for any sample).

**Fig. 3:**
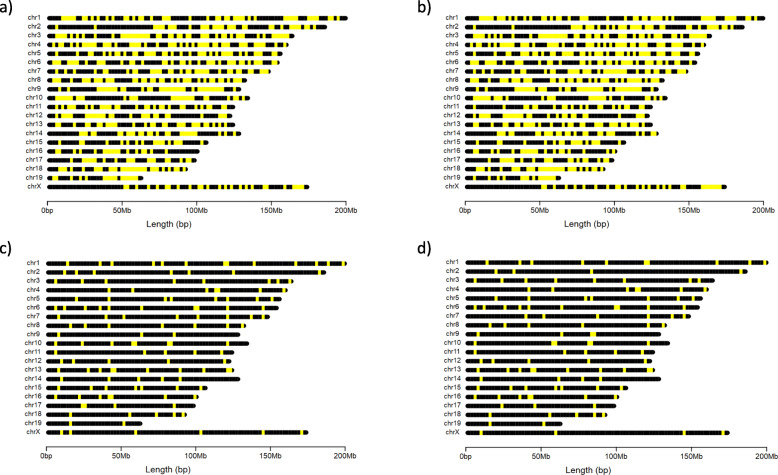
The chromosomal positions in the mouse genome of diagnostic SNPs for backcrosses into C57BL/6J from the following donor strains: **a** BALB/c-AnNHsd: 807 SNPs (**b**) BALB/c-IL4/IL13: 819 SNPs (**c**) C57BL/6N-Crl: 139 SNPs (**d**) C57BL/6N-Hsd: 123 SNPs. Yellow lines indicate the positions of informative SNPs along each chromosome

**Fig. 4:**
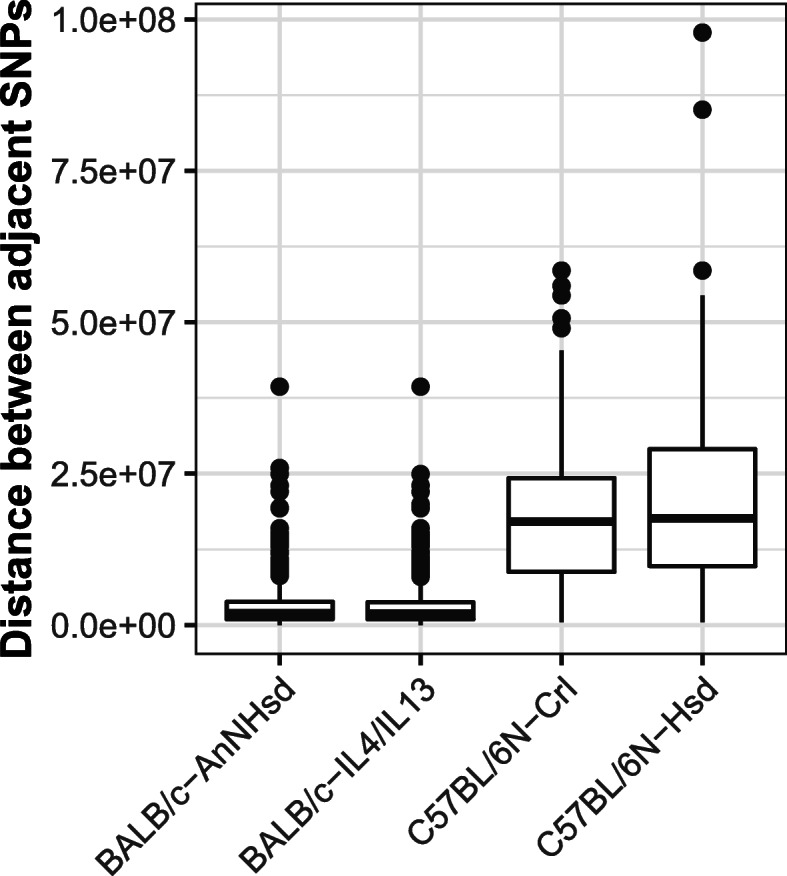
Chromosomal distribution of diagnostic SNPs for backcrosses from donor strains (shown on the x-axis) into C57BL/6J, illustrated by the distribution of distances (number of base pairs) between adjacent SNPs that are divergent between C57BL/6J and the donor strain

After performing filtering steps to identify diagnostic SNPs for each donor strain (assuming the recipient strain is C57BL/6J), we identified 807 diagnostic SNPs for BALB/c-AnNHsd, 819 for BALB/c-IL4/IL13, 139 for C57BL/6N-Crl, and 123 for C57BL/6N-Hsd (Table 3). These diagnostic SNPs were distributed across all chromosomes for each donor strain; BALB/c donor strains had 20–68 SNPs per chromosome and a mean distance between SNPs of 3.01–3.03 Mb, C57BL/6N donor strains had 2–13 SNPs per chromosome and a mean distance between SNPs of 18.9–21.4 Mb (Table 3**,** Figs. 3, 4).

**Table 3:** The number and chromosomal distribution of diagnostic SNPs for backcrosses from four donor strains into C57BL/6J. Min = minimum, Max = maximum

Donor strain	Diagnostic SNPs	Number SNPs per chromosome			Distance between adjacent SNPs (Mb)		
		Mean	Min	Max	Mean	Min	Max
BALB/c-AnNHsd	807	40.4	22	66	3.03	0.0000007	39.4
BALB/c-IL4/IL13	819	41.0	20	68	3.01	0.0000007	39.4
C57BL/6N-Crl	139	6.95	2	13	18.9	0.49	58.6
C57BL/6N-Hsd	123	6.15	2	10	21.4	0.48	97.9

**Fig. 5:**
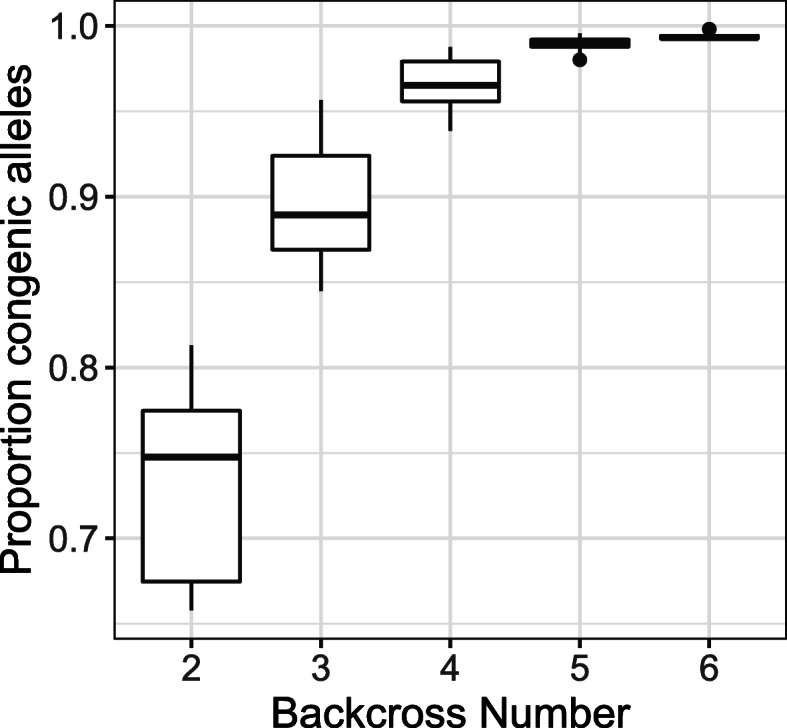
Total proportion of alleles matching the recipient strain for individuals across generations in an experiment backcrossing BALB/c-IL4/IL13 into C57BL/6J. Proportions were calculated using SNPs identified as being diagnostic between the original parent strains (819 SNPs)

For the backcross experiment of BALB/c-IL4/IL13 into C57BL/6J, the percentage of congenic alleles for the 819 diagnostic SNPs increased from a mean of 73.6% (range 65.8–81.3%) in the second backcross to a mean of 99.4% (range 99.3–99.8%) in the sixth backcross (Table 4**,** Fig. 5).

**Table 4:** Sample sizes and mean, minimum, and maximum percentage of recipient strain alleles for backcross individuals from a speed congenic experiment backcrossing BALB/c-IL4/IL13 into C57BL/6J. Percentages are calculated using the SNPs determined to be diagnostic for the two parent strains

Donor strain	Source	Backcross number	***n***	% C57BL/6J alleles		
				Mean	Min	Max
BALB/c-IL4/IL13	Jackson Laboratory (Cat# 015859)	2	13	73.6	65.8	81.3
		3	8	89.6	84.5	95.7
		4	14	96.5	93.9	98.8
		5	28	99.0	98.0	99.6
		6	6	99.4	99.3	99.8

Bioinformatic analyses indicated the mean predicted number of diagnostic SNPs for crosses between each pair of 102 laboratory mouse strains was 549 ± 136 SD, with 95.2% of strain combinations having > 300 diagnostic SNPs (Table S4). These numbers are slightly lower than in [13] because our assay includes a smaller number of SNPs (i.e., our assay uses 1499 of the 1638 SNPs reported in [13]).

## Discussion

Our SNP genotyping assay had consistently high genotyping success rates across samples and across SNPs, with > 94% of SNPs successfully genotyped for > 99% of samples. The assay also had a high genome-wide density of SNPs that were diagnostic for distinguishing the two strains tested (807–819 SNPs distinguishing BALB/c and C57BL/6J). Our backcross experiment of BALB/c into C57BL/6J demonstrated that the assay could be used to generate up to 99.8% congenic offspring within six generations. Furthermore, the assay is predicted to have a high density of diagnostic SNPs for many additional laboratory mouse strains, with a mean of 549 ± 136 SD diagnostic SNPs for crosses between 102 inbred and wild-derived inbred strains, and with 95.2% of strain combinations having > 300 diagnostic SNPs. These densities are much higher than most current speed congenics SNP genotyping platforms, which typically use around 150 diagnostic SNPs per backcross combination. Therefore, our genotyping assay should be highly flexible for a wide variety of backcross strain combinations, and should have a high level of accuracy for characterizing the proportion of the genome that matches the recipient strain. We also demonstrated that our assay has a sufficient density of genome-wide diagnostic SNPs for backcrossing the closely related substrains C57BL/6N and C57BL/6J, which are commonly used in congenics experiments (123–139 SNPs). Although the assay was not explicitly designed for backcrosses between other closely related substrains, the probe set and bioinformatic pipeline can be easily modified to incorporate additional SNPs for custom experiments. Notably, more than 100,000 SNPs can be queried in a single assay with the Allegro Targeted Genotyping approach used here.

Our assay is cost-effective and uses standard laboratory equipment for library prep, making the protocol feasible for most research labs. The total cost of laboratory reagents and consumables for genomic DNA quantification, sample normalization, and library prep is about US$20 per sample. The laboratory work requires about 4 h of hands-on time for sample quantification and normalization of 48 samples, followed by about 8 h for library prep, which includes about 4 h of hands-on time. The library prep can easily be scaled up to a larger number of samples with little increase in hands-on time, thus reducing the time per sample. After library prep, the DNA is sequenced using Illumina MiSeq, HiSeq, MiniSeq, NextSeq, or NovaSeq platforms, which are widely available across core research labs. Here we used MiSeq 2 × 300 sequencing, which typically costs approximately $3000 per lane at core facilities; we reduced the cost of sequencing by using one quarter of a MiSeq lane for each batch of 48 samples, resulting in a per-sample sequencing cost of about US$15. Therefore, the total cost for library prep and sequencing together is about US$35 per sample, which is substantially lower than many speed congenics genotyping service providers.

Another advantage of our assay compared to commercial assays is that it allows the identification of a set of diagnostic SNPs for a backcross experiment without making any prior assumptions about the genomic composition of the original parental strains. Different strains from different sources can have genetic differences; for example, the C57BL/6N samples from two different sources in our study had substantially different numbers of diagnostic SNPs for backcrossing into C57BL/6J (139 diagnostic SNPs for C57BL/6N-Crl from Charles River, and 123 diagnostic SNPs for C57BL/6N-Hsd from Envigo; Table 3). Therefore, a commercial assay designed for a parental strain from one source may not be effective for the same parental strain from another source. In contrast, analysis of the original parental strains with our genotyping assay would allow identification of a set of diagnostic SNPs tailored to a backcross experiment using those parental strains.

We also developed a bioinformatic pipeline which generates clear, pragmatic output for decision-making in speed congenics experiments. This pipeline performs SNP genotyping, sample quality assessment, and calculation of the percentage of alleles matching the recipient strain for each sample. The results of these analyses are used to output a report of the genotypes for each sample and SNP, genotyping success rate across SNPs for each sample, and the number and percentage of alleles that match the recipient strain for each sample. This output allows easy identification and removal of samples with low genotyping performance, selection of the backcross offspring with the greatest percentage ancestry from the recipient strain, and assessment of whether samples are congenic. The pipeline is also fast; analyses were completed with less than 2 h of computational time for 48 samples using a SuperMicro 2028GR-TR server with two Intel Xeon E5–2690 v4 processors (28 physical cores, 56 logical). A description of the bioinformatic pipeline is available at https://github.com/kimandrews/CongenicMouseGenotyping, including step-by-step commands, scripts, a bed file of the target SNPs (this is also in Table S1), and a list of required dependencies.

### Other applications

Although we focus here on the utility of our SNP genotyping assay for developing congenic mice to identify the functions of target genes or mutations, the assay could also be used to address a number of other research questions. For example, the assay could be used to identify and map quantitative trait loci (QTL mapping), conduct standard F2 crosses, and characterize the ancestry of an unknown sample (e.g., forensics) [9, 13, 23]. The assay could also be used to conduct phylogenetic analysis, although the complex breeding history of laboratory mice should be taken into account when interpreting phylogenetic results [13, 24]. The assay also includes 29 Y chromosome markers which could be used for phylogenetic analysis or sex determination for unknown tissue samples. For these and other research questions, our bioinformatic pipeline designed here could be used to generate SNP genotypes for custom downstream analyses.

## Conclusions

We developed a SNP genotyping assay for speed congenics that takes advantage of the power of high throughput sequencing technologies to improve efficiency and affordability. By simultaneously sequencing 1640 informative markers for tens or hundreds of samples, our assay provides a cost-effective, time-efficient, and flexible assay that can be used for many different combinations of backcross strains. The library prep uses standard laboratory equipment, and the DNA sequencing uses Illumina platforms that are found in many core research labs. We also developed a publicly available bioinformatic pipeline that outputs the information required for effectively guiding a speed congenics project, including SNP genotypes and ancestry proportions for each sample. We expect this SNP genotyping assay to be a powerful and practical tool for a wide variety of speed congenics projects.

## Supplementary Information

**Additional file 1: Table S1.** Standard-format “bed” file showing the genomic positions of the SNPs in the mouse genotyping assay. First column = chromosome number, second column = start of the SNP position, third column = end of the SNP position, fourth column = SNP name. **Table S2.** Standard-format “bed” file showing the genomic positions of the probes in the mouse genotyping assay. First column = chromosome number, second column = start of the SNP position, third column = end of the SNP position, fourth column = SNP name, fifth column = score, sixth column = positive or negative DNA strand. **Table S3.** Metadata and genotype summaries for all samples included in the study, including samples used in the experiment comparing three batches of 48 samples (“Experiment1_3Batches”), the experiment identifying diagnostic SNPs for different strains (“Experiment2_Strains”), and the backcross experiment (“Experiment3_Backcross”). Samples included in each of these experiments are indicated by “yes” in the corresponding column; some samples were used in more than one experiment, and some samples were sequenced more than one time as technical replicates, starting from the same genomic DNA extraction (indicated in “ReplicateNumber” column). “LibraryID” = name of the sequencing library; “BatchID” = batch ID for the sequencing run; “Strain” = the strain ID for samples that were not backcrossed individuals; “DonorStrain” = the donor strain for backcrossed individuals; “BackcrossNumber” = backcross number for backcrossed individuals; “BB” = number of SNPs that were homozygous for the alternate allele; “AB” = number of SNPs that were heterozygous for the alternate allele; “AA” = number of SNPs that were homozygous for the reference allele; “failed” = number of SNPs that failed to genotype; “TotalGenotyped” = total number of SNPs successfully genotyped; “ProportionAA” = proportion of genotyped SNPs that were homozygous for the reference allele; “ProportionA” = proportion of reference allele for genotyped SNPs. **Table S4.** Predicted numbers of diagnostic SNPs in the mouse genotyping assay for crosses between each pair of 102 inbred and wild-derived inbred mouse strains. Numbers were calculated based on genotypes reported in [13] for the SNPs that were shared between that study and the assay described here (i.e., *n* = 1499 SNPs).

## Data Availability

Raw Illumina sequence data generated and analyzed for this study are available at the NCBI Short Read Archive (BioProject ID PRJNA723592). Genomic positions of the SNPs and probes in the genotyping assay are provided as standard-format “bed” files in Tables S1 and S2. The Tecan Design ID for the genotyping assay is ST2181G_1. Metadata and genotype summaries for each sample are provided in Table S3. A description of the bioinformatic pipeline is available at https://github.com/kimandrews/CongenicMouseGenotyping, including step-by-step commands, scripts, the bed file of the target SNPs (i.e., the same file as Table S1), and a list of required dependencies.

## Abbreviations

SNP: Single nucleotide polymorphism
QTL: Quantitative trait locus
