# Supplementary material for: A new mouse SNP genotyping assay for speed congenics: combining flexibility, affordability, and power
Source: BMC Genomics. 2021 May 24;22:378. doi: 10.1186/s12864-021-07698-9 (PMC8142480; doi:10.1186/s12864-021-07698-9)
Supplement: Supplementary file 1 — Additional file 1: Table S1. Standard-format “bed” file showing the genomic positions of the SNPs in the mouse genotyping assay. First column = chromosome number, second column = start of the SNP position, third column = end of the SNP position, fourth column = SNP name. Table S2. Standard-format “bed” file showing the genomic positions of the probes in the mouse genotyping assay. First column = chromosome number, second column = start of the SNP position, third column = end of the SNP position, fourth column = SNP name, fifth column = score, sixth column = positive or negative DNA strand. Table S3. Metadata and genotype summaries for all samples included in the study, including samples used in the experiment comparing three batches of 48 samples (“Experiment1_3Batches”), the experiment identifying diagnostic SNPs for different strains (“Experiment2_Strains”), and the backcross experiment (“Experiment3_Backcross”). Samples included in each of these experiments are indicated by “yes” in the corresponding column; some samples were used in more than one experiment, and some samples were sequenced more than one time as technical replicates, starting from the same genomic DNA extraction (indicated in “ReplicateNumber” column). “LibraryID” = name of the sequencing library; “BatchID” = batch ID for the sequencing run; “Strain” = the strain ID for samples that were not backcrossed individuals; “DonorStrain” = the donor strain for backcrossed individuals; “BackcrossNumber” = backcross number for backcrossed individuals; “BB” = number of SNPs that were homozygous for the alternate allele; “AB” = number of SNPs that were heterozygous for the alternate allele; “AA” = number of SNPs that were homozygous for the reference allele; “failed” = number of SNPs that failed to genotype; “TotalGenotyped” = total number of SNPs successfully genotyped; “ProportionAA” = proportion of genotyped SNPs that were homozygous for the reference allele; “ProportionA” = proportion of reference allele [file 12864_2021_7698_MOESM1_ESM.zip › SupplementalTableTitles_20210501.docx]

**Supplemental Table Titles (Tables are in separate files)**

**Table S1.** Standard-format “bed” file showing the genomic positions of the SNPs in the mouse genotyping assay. First column = chromosome number, second column = start of the SNP position, third column = end of the SNP position, fourth column = SNP name.

**Table S2.** Standard-format “bed” file showing the genomic positions of the probes in the mouse genotyping assay. First column = chromosome number, second column = start of the SNP position, third column = end of the SNP position, fourth column = SNP name, fifth column = score, sixth column = positive or negative DNA strand.

**Table S3.** Metadata and genotype summaries for all samples included in the study, including samples used in the experiment comparing three batches of 48 samples (“Experiment1_3Batches”), the experiment identifying diagnostic SNPs for different strains (“Experiment2_Strains”), and the backcross experiment (“Experiment3_Backcross”). Samples included in each of these experiments are indicated by “yes” in the corresponding column; some samples were used in more than one experiment, and some samples were sequenced more than one time as technical replicates, starting from the same genomic DNA extraction (indicated in “ReplicateNumber” column). “LibraryID” = name of the sequencing library; “BatchID” = batch ID for the sequencing run; “Strain” = the strain ID for samples that were not backcrossed individuals; “DonorStrain” = the donor strain for backcrossed individuals; “BackcrossNumber” = backcross number for backcrossed individuals; “BB” = number of SNPs that were homozygous for the alternate allele; “AB” = number of SNPs that were heterozygous for the alternate allele; “AA” = number of SNPs that were homozygous for the reference allele; “failed” = number of SNPs that failed to genotype; “TotalGenotyped” = total number of SNPs successfully genotyped; “ProportionAA” = proportion of genotyped SNPs that were homozygous for the reference allele; “ProportionA” = proportion of reference allele for genotyped SNPs.

**Table S4.** Predicted numbers of diagnostic SNPs in the mouse genotyping assay for crosses between each pair of 102 inbred and wild-derived inbred mouse strains. Numbers were calculated based on genotypes reported in [13] for the SNPs that were shared between that study and the assay described here (i.e., n = 1,499 SNPs).
